# Collagenous colitis in patients treated for cancer: role of immune checkpoint inhibitors. Clinical, histological and immunological features in 15 cases

**DOI:** 10.1111/his.15530

**Published:** 2025-08-06

**Authors:** Mohamed‐Amine Bani, Peggy Dartigues, Ranya Soufan, Simona Cosconea, Michel Ducreux, Caroline Robert, Edouard Guenzi, François‐Xavier Danlos, Aurélien Marabelle, Franck Carbonnel, Jean‐Yves Scoazec

**Affiliations:** ^1^ Department of Pathology Gustave Roussy Villejuif France; ^2^ Department of Digestive Oncology Gustave Roussy Villejuif France; ^3^ Faculté de Médecine Université Paris Saclay Le Kremlin‐Bicêtre France; ^4^ Department of Dermatology Gustave Roussy Villejuif France; ^5^ Department of Pathology Assistance Publique – Hôpitaux de Paris, Hôpital Bichat Paris France; ^6^ Early Drug Development Department Gustave Roussy Villejuif France; ^7^ Department of Gastroenterology Assistance Publique – Hôpitaux de Paris, Hôpital de Bicêtre Le Kremlin‐Bicêtre France

**Keywords:** collagenous colitis, immunotherapy, microscopic colitis

## Abstract

**Background and aims:**

Collagenous colitis is a rare complication of immunotherapy for cancer. We report here 15 cases in order to assess the role of immune checkpoint inhibitors (ICI) in the onset of this disease, describe the histopathological and immunological features of this entity and compare them with ‘idiopathic’ collagenous colitis.

**Methods:**

We retrospectively checked for all diagnoses of collagenous colitis from 2000 to 2024 in our tertiary cancer centre. Clinical charts and the available histopathological material were reviewed. Immunohistochemical profiling was performed to characterize immune cell populations (CD3, CD4, CD8, CD20, CD68, CD163, CD117, PD1 and PD‐L1). A control group of 14 patients without cancer history was included for comparison.

**Results:**

Fifteen cases, diagnosed between 2017 and 2024, were included in the study group. There were nine male and six female, aged 23–81. Fourteen were treated by ICI (anti‐PD1/PD‐L1 in 13 cases, anti‐CTLA4 in 1), 1 by daratumumab. All presented chronic diarrhoea. The typical histological features of collagenous colitis were present: sub‐epithelial band of thickened extracellular matrix, expansion of the lamina propria. The predominant immune cell population was CD8+ T‐lymphocytes in 12 cases. ICI‐treated patients showed higher neutrophilic infiltration, including crypt abscesses and higher macrophage amounts than control patients.

**Conclusion:**

Collagenous colitis in patients treated for cancer is closely associated with the use of anti‐PD1/PD‐L1 antibodies and shows some distinctive characteristics. A further understanding of the consequences of the PD1/PD‐L1 axis blockade may shed further light on the pathogenesis of this rare disease.

## Graphical abstract

Immune checkpoint inhibitor‐associated collagenous colitis exhibits distinct histopathological and immunophenotypic features compared to idiopathic cases, including prominent sub‐epithelial macrophage accumulation and neutrophilic cryptitis. These findings underscore the pathogenic impact of PD1/PD‐L1 axis blockade on colonic mucosa and expand the spectrum of immune‐mediated gastrointestinal toxicity in oncologic immunotherapy.

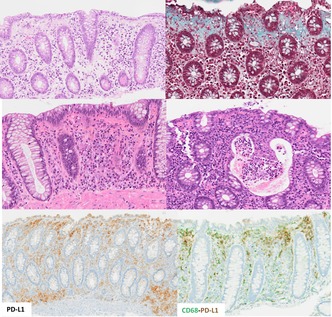

**Abbreviations**
CD: Cluster of Differentiation
CTLA‐4: Cytotoxic T‐Lymphocyte Antigen 4
FFPE: Formalin-Fixed Paraffin-Embedded
GI: Gastrointestinal
ICI: Immune Checkpoint Inhibitor
LAG3: Lymphocyte Activation Gene 3
PD‐1: Programmed Cell Death Protein 1
PD‐L1: Programmed Death Ligand 1
PPI: Proton Pump Inhibitor
SD: Standard Deviation

## Introduction

Collagenous colitis is a form of microscopic colitis characterized by chronic watery, non‐bloody diarrhoea and absence of significant mucosal alterations at endoscopy contrasting with suggestive histopathological features at biopsy, including a sub‐epithelial band of thickened extracellular matrix and an expansion of the immune compartment in the lamina propria.^1^ These histopathological features distinguish collagenous colitis from lymphocytic colitis, the other form of microscopic colitis, characterized by an increase in intra‐epithelial lymphocytes and the absence of a sub‐epithelial collagenous band.^2^ Collagenous colitis and lymphocytic colitis are independent, but related diseases, which may coexist in the same patient, either as mixed lesions or at different times.^2^

The aetiology and pathogenesis of collagenous colitis remain poorly understood. It is usually assumed that collagenous colitis is an immune‐mediated disorder triggered by various stimuli in a predisposed patient, but the mechanisms responsible for the characteristic histopathological lesions are still unclear.^1^, ^3^, ^4^ Various drugs have been associated with an increased risk of collagenous colitis, especially non‐steroidal anti‐inflammatory drugs and proton pump inhibitors, but also serotonin reuptake inhibitors, levodopa/dopa decarboxylase inhibitors, statins and anti‐hypertensive drugs.^1^, ^5^, ^6^ Recently, the immune checkpoint inhibitors (ICI) used in various types of cancer have been added to this list.^7^, ^8^, ^9^ Two main types of ICI are currently used in oncology: anti‐CTLA4 antibodies (such as ipilimumab and tremelimumab) and anti‐PD1/PD‐L1 antibodies (including the anti‐PD1 nivolumab and pembrolizumab and the anti‐PD‐L1 atezolimumab and durvalumab). ICI‐based immunotherapy has proven its efficacy and is gaining large indications in oncology.^10^ However, it also induces numerous side effects,^11^ among which gastrointestinal adverse events are particularly frequent.^8^, ^12^, ^13^ Various types of colitis have been described, including acute colitis, chronic active colitis mimicking inflammatory bowel disease and microscopic colitis, including both lymphocytic colitis and collagenous colitis.^14^

Collagenous colitis has been rarely reported during ICI treatment and several large series of ICI‐related colitis did not include any case.^15^, ^16^, ^17^ However, it raises diagnostic, therapeutic and pathophysiological questions. Some previous studies have suggested that the clinical course of ICI‐related collagenous colitis is more severe than that of conventional collagenous colitis.^13^, ^18^, ^19^ Little information is available on the histological features observed in ICI‐related collagenous colitis and the pathogenic mechanisms involved. So far, it is difficult to address these questions since most cases of collagenous colitis during ICI treatment have been reported as case reports^20^, ^21^, ^22^, ^23^, ^24^ or briefly mentioned in large series describing adverse side effects of these drugs^18^, ^19^, ^25^, ^26^, ^27^, ^28^ Only one small series of ICI‐related collagenous colitis has been described in more detail.^29^ We were, therefore, prompted to report 15 cases of collagenous colitis in patients treated for cancer in our institution in order to: (a) assess the role of ICI in the onset of this disease, (b) describe the histopathological and immunological features of collagenous colitis under ICI treatment and (c) compare them with those of ‘idiopathic’ collagenous colitis.

## Material and Methods

### Study Group

We checked the files of the department of Pathology of our institution, a tertiary reference cancer centre, for all colon biopsies examined between 2000 and 2024. The number of cases with a histologically proven diagnosis of acute, chronic active or microscopic colitis was recorded and for each case, the oncological treatment received at the onset of disease was noted. All cases with a definitive or suspected diagnosis of collagenous colitis and with tissue material available were eligible for study. The following features were recorded from the clinical charts: sex, age at diagnosis, type of cancer, previous history of gastrointestinal disease, treatments of cancer, other medications received at the onset of disease, grade of diarrhoea (according to National Cancer Institute's Common Terminology Criteria for Adverse Events),^30^ other clinical symptoms, microbiological data, delay (in weeks) between treatment initiation and diarrhoea onset, treatment of diarrhoea, response to treatment.

### Histological Study

All the tissue material available in each case, including diagnostic colon biopsies and when present, previous and follow‐up biopsies and samples from other gastrointestinal segments, was reviewed. 4 μm‐thick sections of formalin‐fixed paraffin‐embedded (FPPE) samples were stained with haematoxylin–eosin‐saffron and special stains if necessary (PAS, Alcian blue, Masson trichrome). The thickness of the sub‐epithelial band was measured in well‐oriented sections with an optical micrometre, as previously described.^2^ The following features were evaluated semi‐quantitatively: presence of superficial epithelial injury, characteristics of the sub‐epithelial band (thickness, focal or diffuse distribution), crypt distortion, crypt apoptosis, expansion of immune cells in the lamina propria, presence of basal lymphoplasmocytosis, presence and density of intra‐epithelial lymphocytes, presence, location and abundance (evaluated semi‐quantitatively on a 4‐grade scale) of macrophages, neutrophils and eosinophils.

### Immunohistochemistry

An immunohistochemical study was performed on 3 μm‐thick deparaffinized sections of FPPE tissue samples using an automated device (Ventana Benchmark, Tucson, AZ). The following antibodies were used on serial sections, in the following order: for identification of T‐lymphocytes, CD3 (clone 2GV6; prediluted; Roche, Meylan, France), CD8 (clone SP57; prediluted; Roche) and CD4 (clone SP35; prediluted; Roche); for identification of B‐lymphocytes, CD20 (clone M‐L26; prediluted; Roche); for identification of macrophages, CD68 (clone KP1; prediluted; Roche) and CD163 (clone M‐MRQ‐26; prediluted; Roche); for identification of mast cells, CD117 (clone EP10; prediluted; Roche); for identification of immune checkpoints, PD‐1 (clone NAT105; 1:700 dilution; Abcam, Cambridge, UK) and PD‐L1 (clone SP263; prediluted; Roche). The location of the various immune cell populations in the mucosa was recorded. The density of CD3+, CD4+, CD8+, CD20+, CD117+, PD1+ cells and the area occupied by CD68+ cells were evaluated through image analysis (QuPath^31^ and Halo, Indica Labs, Albuquerque, NM) and expressed, respectively, as the number of cells per surface unit (mm^2^) and as the percentage of tissue surface occupied per surface unit (mm^2^).

In addition, a double labeling CD68 (green)/PD‐L1 (brown) was performed on selected FFPE‐tissue sections using an automated device (Ventana Discovery Ultra). Briefly, after deparaffinization, sections were incubated sequentially with CD68 antibody (clone PG‐M1, Dako, Glostrup, DK), revealed with Discovery Green HRP kit (Roche); then, after heat stripping at pH 6 100°C, with anti‐PD‐L1 antibody (clone SP263), revealed with diaminobenzidine.

### Control Group

Fourteen cases of collagenous colitis from patients without cancer history were included. There were 10 female and 4 male, aged 28–88 (median: 64 years). All had diarrhoea grade 3. None has a history of previous gastrointestinal disease. No chronic use of non‐steroidal anti‐inflammatory drugs or proton pump inhibitors was mentioned for any patient. The same histopathological features as in the study group were recorded. CD68 immunostaining was performed and quantified as above.

### Statistical Analysis

Statistical analysis was performed using chi2‐test and t‐test. A p‐value <0.05 was considered significant.

## Results

### Composition of the Study Group

Nineteen cases of collagenous colitis were retrieved from our files between 2000 and 2024. There were two cases in 2004, one in 2005, one in 2007, one in 2017, two in 2018 and 2019, one in 2020, five in 2021, two in 2022 and one in 2023 and 2024. The cases seen in 2004–2007 were from patients with chronic diarrhoea (*n* = 2, 1 with Lynch syndrome, 1 with colon adenocarcinoma), persistent diarrhoea after surgery for colon adenocarcinoma (*n* = 1) and diarrhoea 2 months after initiation of nivolumab for progressive colon adenocarcinoma following cetuximab and carboplatin (*n* = 1). For these four patients, no tissue material suitable for additional studies was available. Among the 15 cases seen between 2017 and 2024, 14 were from patients treated with ICI. Over this period, this represented 0.45% of all colon biopsies examined in our institution, 1.2% of all cases with a histological diagnosis of acute or chronic colitis and 5.2% of histologically proven cases of colitis in ICI‐treated patients. The remaining case was from a patient with myeloma, treated with daratumumab, an anti‐CD38 monoclonal antibody. In all 15 cases, tissue material was available. These 15 patients were included in the study group.

### Clinical Features

The 14 ICI‐treated patients were 8 male and 6 female, aged from 23 to 81 years (median: 68) (Table 1). None had a history of gastrointestinal disease or chronic diarrhoea prior to ICI use. Indications for treatment were: squamous cell carcinoma (head and neck, *n* = 3; uterine cervix, *n* = 1; skin, *n* = 1), melanoma (*n* = 2), lung adenocarcinoma (*n* = 2), gastric adenocarcinoma (*n* = 1), urothelial carcinoma (*n* = 1), hepatocellular carcinoma (*n* = 1), liposarcoma (*n* = 1), anaplastic lymphoma (*n* = 1). Seven patients were metastatic, five had locally advanced disease and three were relapsing. Previous treatments included: chemotherapy in nine patients, targeted therapy (b‐RAF inhibitor) in one patient, anti‐angiogenic therapy in two patients; two patients received no previous medical therapy.

**Table 1:** Clinical features

	Sex	Age at onset (years)	Tumour	History of GI disease	Previous treatments	Immunotherapy (IT)	Proton pomp inhibitor	Delay of onset (weeks)	Diarrhoea grade	Other symptoms	Treatment	Duration of treatment (days)	IT resumed
Patient 1	F	70	Lung adenocarcinoma RAS mutated	No	CT	Nivolumab	Omeprazole	26	4	Abdominal pain	Methylpred	15	No
Patient 2	M	72	Gastric carcinoma dMMR	No	CT	Tremelimumab + aOX40	Lansoprazole	7	3	Nausea, vomiting, fever	Methylpred	5	No
Patient 3	F	65	Upper tract urothelial carcinoma	No	CT	Atezolizumab	No	16	2	Abdominal pain	Prednisone	30	No
Patient 4	M	52	Melanoma b‐RAF mutated	No	TT	Pembrolizumab	Lansoprazole	20	3	Abdominal pain, nausea, rectal bleeding	Prednisone	30	No
Patient 5	M	66	Squamous cell carcinoma, tonsil	No	CT	Nivolumab	No	19	2	No	Prednisone	23	Yes
Patient 6	M	78	Squamous cell carcinoma, oropharynx, p16+	No	CT	Nivolumab	Lansoprazole	23	2	Abdominal pain	Methylpred	20	No
Patient 7	F	81	Squamous cell carcinoma, oral	No	CT	Nivolumab	Lansoprazole	8	3	No	Loperamide	15	No
Patient 8	M	70	Melanoma	No	No	Spartalizumab +antiLAG3	Lansoprazole	2	2	No	Budesonide	25	Yes
Patient 9	M	61	Dedifferentiated liposarcoma	No	CT	Nivolumab +Relatlimab	No	25	2	No	Budesonide	25	Yes
Patient 10	F	25	Anaplastic lymphoma ALK+	No	CT	Nivolumab	Lansoprazole	18	2	Rash	Prednisone	7	Yes
Patient 11	F	78	Squamous cell carcinoma, skin	No	No	Pembrolizumab	No	48	2	No	Loperamide	7	Yes
Patient 12	M	65	Lung adenocarcinoma RAS mutated	No	CT	Pembrolizumab	Omeprazole	30	3	No	Methylpred	45	Yes
Patient 13	M	78	Hepatocarcinoma	No	aA	Attezolimumab	Lansoprazole	8	3	No	Prednisone	75	Yes
Patient 14	F	65	Squamous cell carcinoma, uterine cervix	No	aA	Pembrolizumab	No	8	3	No	Methylpred	7	Yes
Patient 15	M	48	Myeloma, IgG lambda	No	No	Daratumumab	No	31	3	No	Prednisone	15	—

aA, anti‐angiogenic therapy; CT, chemotherapy; F, female; GI, gastrointestinal; M, male; methylpred, intravenous methylprednisolone; TT, targeted therapy.

One patient, with gastric adenocarcinoma, received anti‐CTLA4 tremelimumab combined with an OX40 agonist (clinical trial NCT02705482, described in (29)). Eleven patients received anti‐PD1 therapy: nivolumab (*n* = 6), pembrolizumab (*n* = 4) or spartalizumab (*n* = 1); in two cases, nivolumab and spartalizumab were combined with an anti‐LAG3 antibody. Two patients received anti‐PD‐L1 atezolimumab. Nine patients were treated with proton pump inhibitors (lansoprazole, *n* = 7; omeprazole, *n* = 2). All patients had diarrhoea, grade 2 (*n* = 7), grade 3 (*n* = 6) or grade 4 (*n* = 1). Four patients had abdominal pain, two had nausea and vomiting, and one had rectal bleeding. The delay of diarrhoea onset after ICI initiation ranged from 2 to 48 weeks (median: 18.5). No patient had evidence of bacterial (including *Clostridioides difficile* toxin research), viral or parasitic infection at stool and serological examinations. In all patients, colonoscopy showed no significant mucosal alteration. The treatment of diarrhoea was: loperamide in two cases, budesonide in two, prednisone in five and intravenous methylprednisolone in five. Duration of treatment ranged from 5 to 75 days (median: 20). No complication was observed. No second‐line treatment was required. Immunotherapy was resumed in eight patients.

The remaining case was from a 48‐year‐old male patient receiving daratumumab, an anti‐CD38 antibody, for myeloma; grade 3 diarrhoea started 31 weeks after treatment initiation and required prednisone therapy.

### Histological Features

The key diagnostic features of collagenous colitis were present in all patients. The characteristic sub‐epithelial band of dense extracellular matrix, with a thickness > 10 μm and typically engulfing small capillaries, was continuous in 10 patients (9 ICI‐treated patients and the daratumumab‐treated patient) and discontinuous in 5 ICI‐treated patients. An increase in immune cells, including lymphocytes and plasma cells, in the lamina propria was present in all 15 patients; there was no basal lymphoplasmacytosis in any patient.

**Figure 1:**
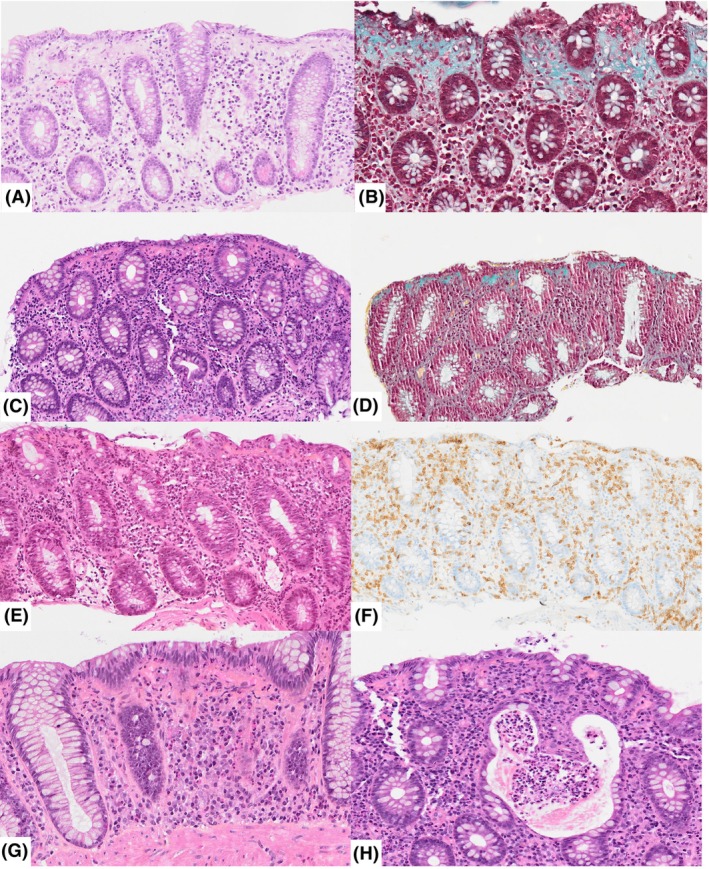
Histopathological features. A typical aspect of collagenous colitis is shown in panels (**A**) and (**B**), with a thick sub‐epithelial band of dense extracellular matrix, highlighted by Masson trichrome (**B**), engulfing small capillaries (open arrowheads); the inflammatory infiltrate in the lamina propria is rich in plasma cells (arrowheads), along with lymphocytes, neutrophils (thin arrows) and macrophages (open arrows); there is no intra‐epithelial lymphocytosis. In panels (**C**) and (**D**), the sub‐epithelial collagenous band is discontinuous (open arrowheads) but well visible in Masson trichrome, with the engulfing of a few capillary vessels; clusters of eosinophils are visible (thin arrows); neutrophilic cryptitis is present (open arrows). Panels (**E**) and (**F**) illustrate a mixed form, combining the presence of a sub‐epithelial collagenous band (open arrowheads) with a dense intra‐epithelial lymphocytosis, highlighted by CD3 immunohistochemistry (**F**); note the presence of scattered eosinophils (thin arrows). In panel (**G**), apoptotic bodies are present within the surface epithelium (thin arrows) above the collagenous band (open arrowheads); severe crypt distortion is visible; the inflammatory infiltrate is rich in plasma cells and eosinophils. Panel (A) illustrates a large neutrophilic crypt abscess (open arrows). A, C, E, G, H: haematoxylin–eosin‐saffron staining; (**B**, **D**) Masson trichrome; (**F**) immunoperoxidase with nuclear counterstaining.

Epithelial lesions were frequent. Surface epithelial injury was present in 10 ICI‐treated patients and in the daratumumab‐treated patient. Crypt distortion was observed in three ICI‐treated patients and intra‐cryptic epithelial apoptosis in two ICI‐treated patients (Table 2 and Figure 1).

**Table 2:** Histological findings

	Study group															Control group
	1	2	3	4	5	6	7	8	9	10	11	12	13	14	15	*N* = 14
Treatment	ICI	ICI	ICI	ICI	ICI	ICI	ICI	ICI	ICI	ICI	ICI	ICI	ICI	ICI	DAR	−
Proton pump inhibitor	Yes	Yes	No	Yes	No	Yes	Yes	Yes	No	Yes	No	Yes	Yes	No	No	−
Surface epithelial injury	Yes	Yes	Yes	Yes	Yes	Yes	No	Yes	Yes	Yes	No	No	No	Yes	Yes	Yes: 1/14
Sub‐epithelial band	C	D	C	C	D	C	C	D	C	D	D	C	C	C	C	C: 10; D: 4
Crypt distortion	No	Yes	Yes	No	Yes	No	Yes	No	No	No	No	Yes	No	No	No	Yes: 0/14
Increased epithelial cell apoptosis	No	No	No	No	No	No	No	No	No	Yes	Yes	No	No	No	No	Yes: 0/14
Expansion of lamina propria	Yes	Yes	Yes	Yes	Yes	Yes	Yes	Yes	Yes	Yes	Yes	Yes	Yes	Yes	Yes	Yes: 14/14
Basal lymphoplasmocytosis	No	No	No	No	No	No	No	No	No	No	No	No	No	No	No	Yes: 0/14
Increased intra‐epithelial lymphocytes	No	No	No	No	Yes	No	Yes	No	Yes	No	No	No	No	No	No	Yes: 0/14
Presence of macrophages	++	+	++	++	+	+	+	+	++	+++	+	+	++	+	+	
Neutrophilic cryptitis	Yes	Yes	Yes	Yes	Yes	Yes	Yes	Yes	Yes	Yes	Yes	Yes	Yes	No	No	Yes: 5/14
Neutrophilic abcesses	No	Yes	Yes	No	No	No	No	No	No	Yes	No	No	No	No	No	Yes: 0/14
Presence of eosinophils	−	++	++	−	++	+	+	+	++	+	+++	+	+	+	++	

C, continuous; D, discontinuous; DAR, daratumumab; ICI, immune checkpoint inhibitor.

Intra‐epithelial lymphocytosis (>20 lymphocytes/100 surface epithelial cells) was present in three ICI‐treated patients. In addition to lymphocytes and plasma cells, other immune cells were present in the immune infiltrate. Macrophages were identifiable in low (*n* = 9), moderate (*n* = 5) or high (*n* = 1) numbers in all patients; they were usually more numerous immediately below the sub‐epithelial collagenous band. Neutrophilic cryptitis was present in 13 out of the 14 ICI‐treated patients; crypt abscesses were observed in 3 cases. Eosinophils were detected in 12 ICI‐treated patients and in the daratumumab‐treated patient; they were scattered in the lamina propria in low (*n* = 7), moderate (*n* = 5) or high (*n* = 1) numbers.

In one patient (#5), previous colon biopsies, performed 6 months earlier, showed features compatible with lymphocytic colitis, characterized by intra‐epithelial lymphocytosis without thickening of the sub‐epithelial matrix. No evidence of involvement of other gastrointestinal segments was found in any patient.

### Immunological Features

**Figure 2:**
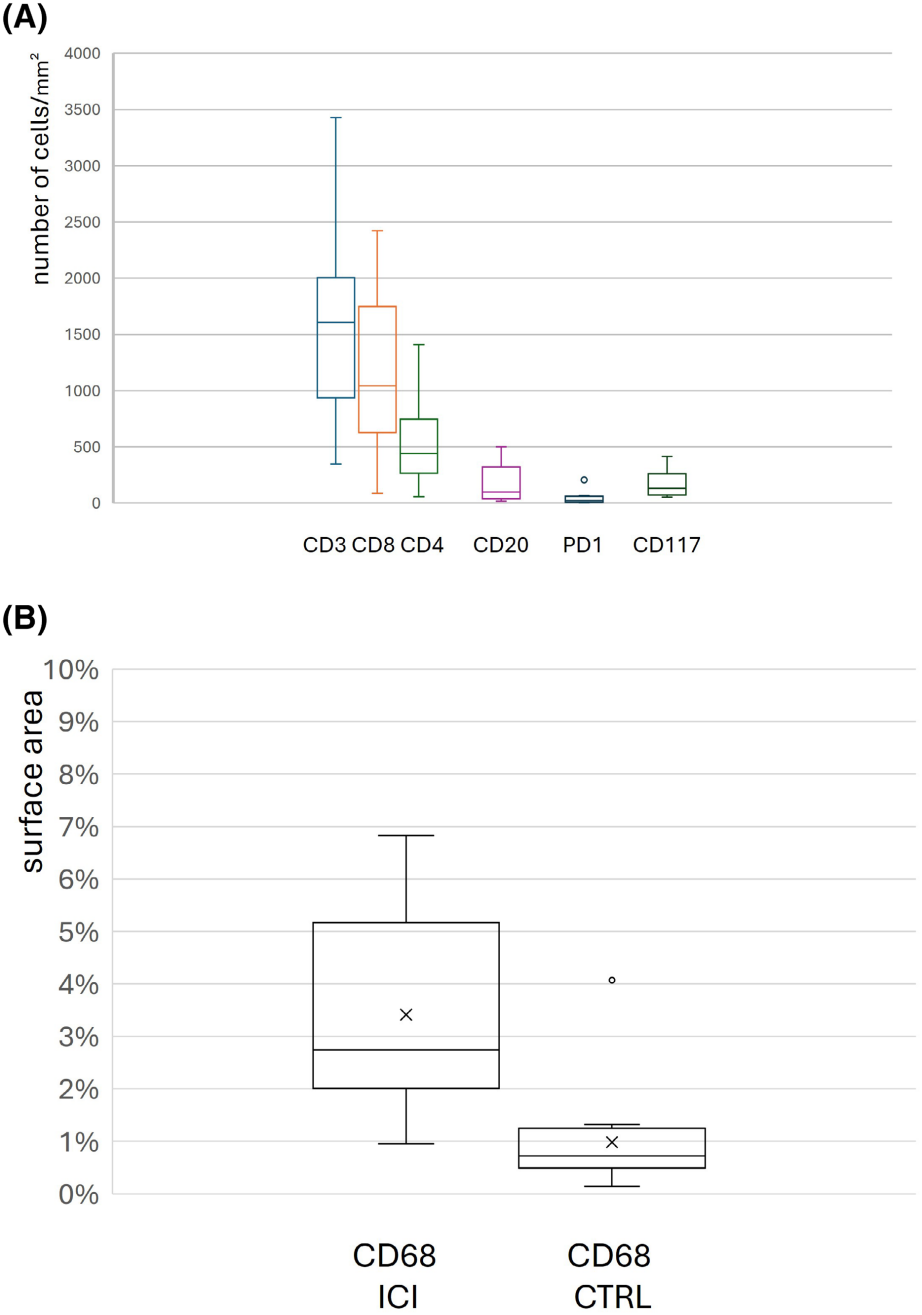
Characteristics of the immune infiltrates. In (**A**), are represented the distributions of cell densities for, respectively, CD3+, CD8+, CD4+, CD20+, PD1+, CD117+ cells in the study group. In (**B**), is represented the tissue surface area occupied by CD68+ macrophages in ICI‐treated patients (CD68 ICI) as compared to the control group (CD68 CTRL).

**Figure 3:**
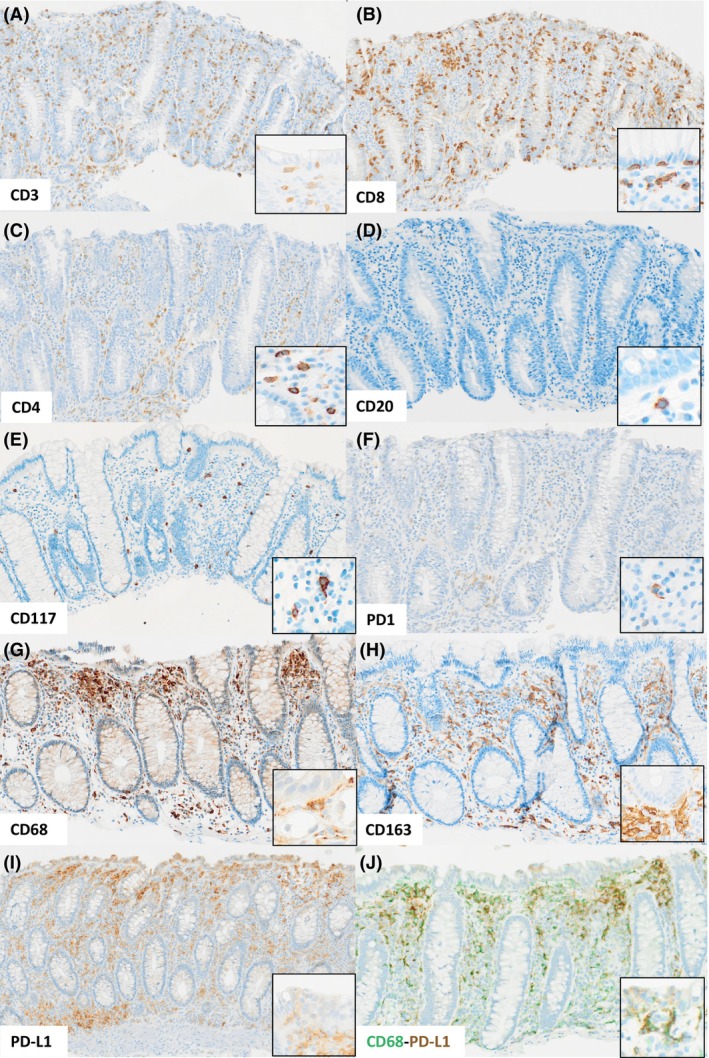
Immunohistochemical features. Representative immunostainings for the T‐cell markers CD3 (**A**), CD8 (**B**), CD4 (**C**), the B‐cell marker CD20 (**D**), the mast‐cell marker CD117 (**E**), PD1 (**F**), the macrophage markers CD68 (**G**) and CD163 (**H**), PD‐L1 (**I**); a double labeling for CD68 (green) and PD‐L1 (brown) is shown in (**J**). Note the sub‐epithelial accumulation of CD68+ macrophages (**G**) which also express PD‐L1 (**H** and **J**) and CD163 (**I**). For each marker, a high‐power insert highlights representative positive cells. Immunoperoxidase with nuclear counterstaining.

The immune cell population contained predominantly CD3+ T‐lymphocytes (range: 347–3426 cells/mm^2^; mean ± SD: 1649 ± 814), while CD20+ B‐lymphocytes were usually in low numbers (range: 19–501 cells/mm^2^; mean ± SD: 172 ± 154) (Figures 2 and 3). Intra‐epithelial lymphocytes were CD3+ CD8+. Among mucosal T‐lymphocytes, CD8+ cells (range: 89–2422 cells/mm^2^; mean ± SD: 1173 ± 711) were usually more numerous than CD4+ cells (range: 59–1403 cells/mm^2^; mean ± SD: 441 ± 579). The proportion of CD8+ cells in the T‐cell infiltrates ranged from 60 to 90% in 12 ICI‐treated patients (median: 76.7%); CD4+ cells predominated in 3 cases: 2 ICI‐treated patients (#13 and #14, respectively, 54 and 74% of T cells), and the daratumumab‐treated patient (66% of T cells). PD1+ cells were rare or very rare, except in 2 ICI‐treated patients (#5 and #14) (range: 4–211 cells/mm^2^; mean ± SD: 49 ± 68). PD‐L1 was expressed by immune cells, macrophages and rare epithelial cells. CD117+ mast cells were present in variable numbers (range: 54–416 cells/mm^2^; mean ± SD: 178 ± 125). CD68+ macrophages were usually abundant and mainly located within the sub‐epithelial collagenous band (range of percentage of tissue surface occupied: 1–7%; mean ± SD: 3.4% ± 2); most of them were CD163+ and PD‐L1+.

### Comparison with the Control Group

The typical sub‐epithelial collagen band was continuous in nine control cases and discontinuous in five; there was no significant difference with the study group.

Epithelial injury was more severe in the study group than in the control group. Surface epithelial injury was present in 10 ICI‐treated cases (71%), compared to 1 case (7%) in the control group (*χ* ^2^ test, *P* < 0.0005). Crypt distortion was observed in 5 ICI‐treated cases (36%) and in none of the control cases (*χ* ^2^ test, *P* < 0.02). Increased epithelial apoptosis was noted in two ICI‐treated cases (13%) and in none of the controls (*χ* ^2^ test, *P* > 0.05).

Expansion of the lamina propria was constantly seen in both groups. Increased intra‐epithelial lymphocytes were found in three ICI‐treated cases (20%) and in none of the controls. Neutrophilic cryptitis was present in 13 ICI‐treated cases (93%) and in 5 control cases (36%) (*χ* ^2^ test, *P* < 0.05). Neutrophilic crypt abscesses, observed in 3 ICI‐treated cases, were detected in none of the control cases. Eosinophils were detected in both groups, with no statistically significant difference.

Macrophages were present in all cases in both groups. CD68+ cells were significantly more numerous in ICI‐treated patients (range of percentage of tissue surface occupied: 1%–7%; mean ± SD: 3.4% ± 2%) than in the control group (range of percentage of tissue surface occupied: 0.1%–4.1%; mean ± SD: 1 ± 0.96) (*t*‐test, *P* < 0.05).

### Clinicopathological Correlations

There was no significant difference in histological features according to PPI use and type of ICI. We observed no statistically significant correlation between the density of the various immune cell populations (neutrophils, CD3, CD8, CD4, macrophages) and the following features: sex, PPI use, grade of diarrhoea, type of ICI (nivolumab vs. pembrolizumab), response to treatment. The density of CD8+ and CD4+ cells tended to decrease with the grade of diarrhoea (respectively, 1531 ± 577 CD8+ cells/mm^2^ and 709 ± 493 CD4+ cells/mm^2^ in grade 2 diarrhoea versus 661 ± 389 and 356 ± 216 in grade 3 diarrhoea) but the difference was not statistically significant. In the same way, the densities of CD8+ and CD4+ cells tended to be lower in patients taking PPI than in patients without PPI use (respectively, 715 ± 657 CD8+ cells/mm^2^ vs. 1210 ± 826 and 392 ± 464 CD4+ cells/mm^2^ versus 514 ± 426) but the differences were not statistically significant.

## Discussion

In our experience, in patients treated for cancer, collagenous colitis is rare and closely, while not exclusively, associated with ICI administration and among ICI, with anti‐PD1/PD‐L1 antibodies. Our results suggest that ICI‐related collagenous colitis is heterogeneous in its histopathological and immunological features and presents some distinctive characteristics as compared to ‘idiopathic’ collagenous colitis occurring in patients without cancer history.

Only 19 newly diagnosed cases of collagenous colitis were retrieved over a 25‐year period in our institution, a tertiary cancer reference centre; while our series is, by far, the largest reported in the literature, the relatively low number of cases available, because of the rarity of the disease, is one of the limitations of our work. The rarity of the disease is confirmed by other studies: in a very large series of microscopic colitis in cancer patients observed over a 7‐year period, only two cases of collagenous colitis were included.^18^ The striking majority of our cases (16 out of 19) were observed in the immunotherapy era and were associated with ICI treatment. Only one case was observed with another drug, the anti‐CD38 antibody daratumumab, which is known to induce diarrhoea^32^ and is reported here for the first time associated with collagenous colitis. Among ICI‐treated patients with diarrhoea and histologically proven colitis seen in our institution, the incidence of collagenous colitis was 5.2% over the study period. This is comparable to the incidence in the general population of patients explored for chronic diarrhoea, estimated at 4.96%.^1^ Despite the increasing use of ICI‐based immunotherapy in cancer patients, we did not observe a corresponding increase in the number of cases of collagenous colitis in our institution: the sharp increase observed in 2021 as compared to the previous years was not confirmed in the following years. This may indicate a better management of patients.

Among ICI, anti‐PD1/PD‐L1 antibodies are, by far, the most frequently incriminated. This was the case in 14 of our patients (among which 13 included in our study group) and in all the cases so far reported in the literature. Almost all anti‐PD1/PD‐L1 antibodies have been involved. As in our series, the largely used anti‐PD1 nivolumab and pembrolizumab are the most frequently cited in the literature.^18^, ^19^, ^20^, ^22^, ^23^, ^27^, ^29^ We report here the first case with another, more recent, anti‐PD1 antibody, spartalizumab. It must be noted that, in two patients, anti‐PD1 antibodies were used in combination with an anti‐LAG3 antibody, which may also induce, while rarely, diarrhoea and colitis.^33^ Cases with the anti‐PD‐L1 atezolimumab^24^ and durvalumab^29^ have also been reported in the literature; two patients under atezolimumab were included in our study group.

In our series as well as in the literature, no case has been observed during treatment with the largely used anti‐CTLA4 antibody ipilimumab, even if some patients had received the drug before,^20^ or in combination with,^18^, ^19^, ^29^ anti‐PD1/PD‐L1 immunotherapy. One of our patients received another anti‐CTL4 antibody, tremelimumab, but its role is difficult to assess; the drug was associated with an OX40 agonist: even if the adverse effects of this compound are not well known, OX40 stimulation is known to be experimentally colitogenic^34^; moreover, the patient also received daily lansoprazole: this proton pump inhibitor is definitely associated with an increased risk of collagenous colitis.^5^ Even if the reporting of medications taken for other reasons than cancer treatment is unlikely to be exhaustive in the clinical charts of the patients included in our study, at least eight other patients of our study group also received proton pump inhibitors. This raises the possibility that, at least in some patients, ICI‐associated collagenous colitis could be the exacerbation of a preexisting, unrecognized or asymptomatic disease, as documented in some studies^35^ or may be favoured by the association with other medications, such as proton pump inhibitors, as previously underlined.^29^

The cases of ICI‐associated collagenous colitis collected in our series presented all the key diagnostic features of the disease. Clinically, chronic watery diarrhoea was constantly present, while of various grades, from mild to severe; other symptoms were rare and severe complications were not observed. All patients required treatment, usually with corticosteroids, including budesonide, prednisone or prednisolone; no second‐line treatment was required. Histologically, the typical lesions,^2^ including the sub‐epithelial thickening of the extracellular matrix and the expansion of the immune compartment in the lamina propria consisting mainly of lymphocytes associated with plasma cells, were present. In five cases, the sub‐epithelial collagenous band was only discontinuous. Interestingly, focal or segmental sub‐epithelial thickening of the extracellular matrix has been frequently noted in some series of ICI‐associated colitis, even in cases resembling other types of inflammatory bowel disease.^28^ We observed a certain degree of heterogeneity in the composition and distribution of the immune infiltrates. Intra‐epithelial lymphocytosis was present in three cases, suggesting a mixed form of microscopic colitis^2^: indeed, pure lymphocytic colitis was diagnosed in the previous biopsies of one of these patients. Macrophages were present in variable numbers, usually within the sub‐epithelial compartment. Eosinophils and mast cells were inconstantly present, but could be observed in significant numbers. Immunologically, the landscape was variable. Most cases were rich in CD8+ T‐lymphocytes and poor in CD20+ B‐lymphocytes, as expected in collagenous colitis^36^, ^37^ but also as described in anti‐PD1/PD‐L1‐related colitis.^16^, ^38^, ^39^ However, CD4+ T‐lymphocytes predominated in two cases of ICI‐associated collagenous colitis. PD1 was expressed only by rare lymphocytes scattered in the lamina propria; PD‐L1 was detected on immune cells, mainly macrophages, in the lamina propria and also on some epithelial cells. As previously reported in conventional collagenous colitis,^37^, ^40^, ^41^ we could not find any correlation between clinical features and histopathological or immunological characteristics.

While our cases of ICI‐associated collagenous colitis present many typical features of the disease, they also show some peculiarities, most of them shared with other types of ICI‐related colitis. Clinically, as in a previous series,^29^ we did not observe the strong female predominance usually described in collagenous colitis^1^, ^42^ and that we observed in the control group; on the contrary, like the previous one, our series shows a slight male predominance. Histologically, a distinctive feature observed in our study was the almost constant presence of abundant neutrophils, resulting in neutrophilic cryptitis in most patients and even crypt abscesses in three cases. Neutrophils are usually rare in conventional collagenous colitis,^2^, ^41^ as verified in our control group, but are frequently observed in anti‐PD1/PD‐L1‐related colitis.^15^ In addition, high numbers of CD68+ macrophages were observed in our cases; they typically accumulated in the sub‐epithelial compartment, under the collagen band and retained the phenotype of normal mucosal intestinal macrophages, including the expression of CD163 and PD‐L1.^43^ The presence of sub‐epithelial macrophages has been previously noted in collagenous colitis^44^ as well as in ICI‐related colitis^45^ but their role has been little explored despite their importance in gut homeostasis and response to injury.^46^ It is not possible to determine whether these differences are due to different pathogenic mechanisms or if they correspond to different stages of the disease, ICI‐treated patients being likely biopsied earlier than patients without treatment.

The pathogenic mechanisms involved in the only case of daratumumab‐related collagenous colitis observed in our study might be related. Daratumumab targets not only CD38+ tumour cells, such as myeloma cells, but also normal CD38+ immunosuppressive B, T and myeloid cells and induces, like anti‐PD1/PD‐L1 therapy, an expansion of activated cytotoxic T cells.^47^, ^48^ Interestingly, CD38 targeting has been proposed as a strategy to overcome anti‐PD1/PD‐L1 resistance, mainly through the impairment of the terminal differentiation of exhausted T cells.^49^, ^50^

In conclusion, collagenous colitis in cancer patients is closely associated with anti‐PD1/PD‐L1 antibodies. ICI‐related collagenous colitis mimics conventional collagenous colitis but shows some peculiarities shared with the other types of ICI‐related colitis. It is not therefore a true model for collagenous colitis, but the very strong association with PD1/PD‐L1 blockade suggests that a further understanding of the impact of the blockade of this axis, not only on T cells^51^, ^52^ but also on other immune cells, macrophages and mesenchymal cells—may shed further light on the pathogenesis of this rare disease.^4^, ^9^

## Author contributions

M.A.B.—conception of the study, data collection and analysis and writing and editing of the manuscript. P.D., R.S., S.C., M.D., C.R., E.G., F.X.D., A.M., F.C.—contribution to data collection and analysis, critical review of the manuscript and final approval. J.Y.S.: conception and design of the study, data collection and analysis and writing and editing of the manuscript.

## Funding information

No funding.

## Conflicts of interest

No conflict of interest.

## Data Availability

The data underlying this article will be shared on reasonable request to the corresponding author.
